# Benefits and Caveats in the Use of Retinal Pigment Epithelium-Specific Cre Mice

**DOI:** 10.3390/ijms25021293

**Published:** 2024-01-20

**Authors:** Sai Kocherlakota, Myriam Baes

**Affiliations:** Laboratory of Cell Metabolism, Department of Pharmaceutical and Pharmacological Sciences, KU Leuven, 3000 Leuven, Belgium

**Keywords:** retinal pigment epithelium, knockout, mouse, Cre mice

## Abstract

The retinal pigment epithelium (RPE) is an important monolayer of cells present in the outer retina, forming a major part of the blood–retina barrier (BRB). It performs many tasks essential for the maintenance of retinal integrity and function. With increasing knowledge of the retina, it is becoming clear that both common retinal disorders, like age-related macular degeneration, and rare genetic disorders originate in the RPE. This calls for a better understanding of the functions of various proteins within the RPE. In this regard, mice enabling an RPE-specific gene deletion are a powerful tool to study the role of a particular protein within the RPE cells in their native environment, simultaneously negating any potential influences of systemic changes. Moreover, since RPE cells interact closely with adjacent photoreceptors, these mice also provide an excellent avenue to study the importance of a particular gene function within the RPE to the retina as a whole. In this review, we outline and compare the features of various Cre mice created for this purpose, which allow for inducible or non-inducible RPE-specific knockout of a gene of interest. We summarize the various benefits and caveats involved in the use of such mouse lines, allowing researchers to make a well-informed decision on the choice of Cre mouse to use in relation to their research needs.

## 1. Introduction

The retina can be broadly divided into two major components: the neural retina, consisting of neuronal cells, like photoreceptors, interneurons, glial cells, astrocytes and ganglion cells; and a non-neural retinal pigment epithelium (RPE). The RPE is a monolayer of post-mitotic hexagonally-shaped pigmented cells, interconnected by tight junctions [1,2]. It forms an integral part of the blood–retina barrier (BRB), regulating the transport of nutrients and ions in and out of the underlying neural retina [1,2,3,4].

While it is the neural retina that is responsible for the generation and propagation of the nerve signal in response to a light stimulus, the RPE plays an essential role in the maintenance of the structural and functional integrity of the neural retina [1,3]. This involves carrying out various functions, including the daily phagocytosis and recycling of damaged photoreceptor outer segments, the regeneration of visual chromophore 11-cis-retinal required for phototransduction, the immune regulation of the retina, and the secretion of growth factors [3]. Consequently, any defects in the RPE often result in degeneration of the underlying neural retina [5,6,7]. Indeed, sporadically occurring age-related macular degeneration (AMD), the leading cause of blindness among the elderly in developed nations [8], and many less common inherited retinal disorders such as Stargardt’s disease and Leber congenital amaurosis, are thought to originate in the RPE [9,10]. To efficiently carry out diverse functions such as forming a barrier and intensive phagocytosis, the RPE has evolved unique features, which remain poorly studied. Therefore, it is becoming increasingly important that the cell biology of the RPE and the role of various genes within these cells be well understood.

RPE biology can be studied via both in vitro and in vivo approaches. Various in vitro models including primary RPE cells and differentiated pluripotent stem cells have successfully been used to reveal deregulations upon genetic mutations [11,12], and to study RPE pathology during AMD [1,13]. Stem cell-derived 3D retinal organoids could also potentially provide a powerful tool in such endeavors. However, these procedures are limited by the need for a very specific expertise in culture and differentiation of RPE cells in vitro [1,13] and by a current inability to obtain RPE cells surrounding the photoreceptor cells in 3D retinal organoids [14]. Moreover, owing to the close interactions between the RPE and the photoreceptors, it remains necessary to investigate RPE cells in their native environment [1,3,7,15]. To this end, the RPE has been studied in many mouse models with gene knockouts. However, both the intricate interplay between photoreceptors and the RPE [1,3,15], and influences on the RPE due to the systemic loss of the gene, hinder the elucidation of the role of a particular protein in the RPE. Therefore, mice with an RPE-specific knockout of a gene of interest are required.

Studying RPE-specific knockout mice has additional advantages. Firstly, they are useful when studying the role of a gene that is lethal when knocked out globally, as it may be essential for the development of the organism [16], such as the various peroxisome biogenesis genes [17] and autophagy-related genes [18]. Secondly, the effect of a gene knockout specifically in the RPE on the rest of the retina can be studied. Finally, some of these RPE-specific knockout mice allow for an inducible knockout of the gene of interest, enabling a researcher to study the importance of the gene at various stages in the life cycle of the mouse.

All RPE-specific gene knockout mice to date employ the famed Cre-LoxP system [19,20]. In this review, the various Cre mice available today that specifically target the RPE will be discussed, with their benefits and caveats. These mice will be compared with regard to features like the specificity, timing, inducibility and potential toxicity of Cre expression, allowing researchers to make a well-informed decision about the right Cre mice to use in order to answer their specific research question.

## 2. The Cre-LoxP System

The Cre-LoxP system involves the use of mice expressing Cre recombinase, a site-specific recombinase derived from phage P1, which specifically recognizes a partially palindromic target sequence called LoxP [19,20]. These LoxP sites are constituted of two 13 bp repeats and a central 8 bp unique spacer region which determines its orientation. Cre recombinase is capable of excising sequences flanked by two LoxP sites (floxed) in the same orientation, allowing for genetic knockout of any gene of interest. In the Cre-LoxP system, the promotor driving the expression of Cre recombinase is the crucial factor determining the spatial and temporal inactivation of the gene of interest. In this case, an RPE-specific knockout of a particular gene can be achieved by driving the expression of Cre recombinase through promoters of genes that are specifically expressed in RPE cells. This typically leads to the expression of Cre recombinase with the same spatio-temporal expression pattern as that of the chosen gene.

Another level of control over Cre recombinase expression or function can be achieved with the use of inducible Cre-LoxP systems, allowing for a temporally regulated knockout of the gene of interest. This is most commonly carried out using either the tamoxifen-inducible system or the tetracycline- or doxycycline-inducible system [19].

**Figure 1:**
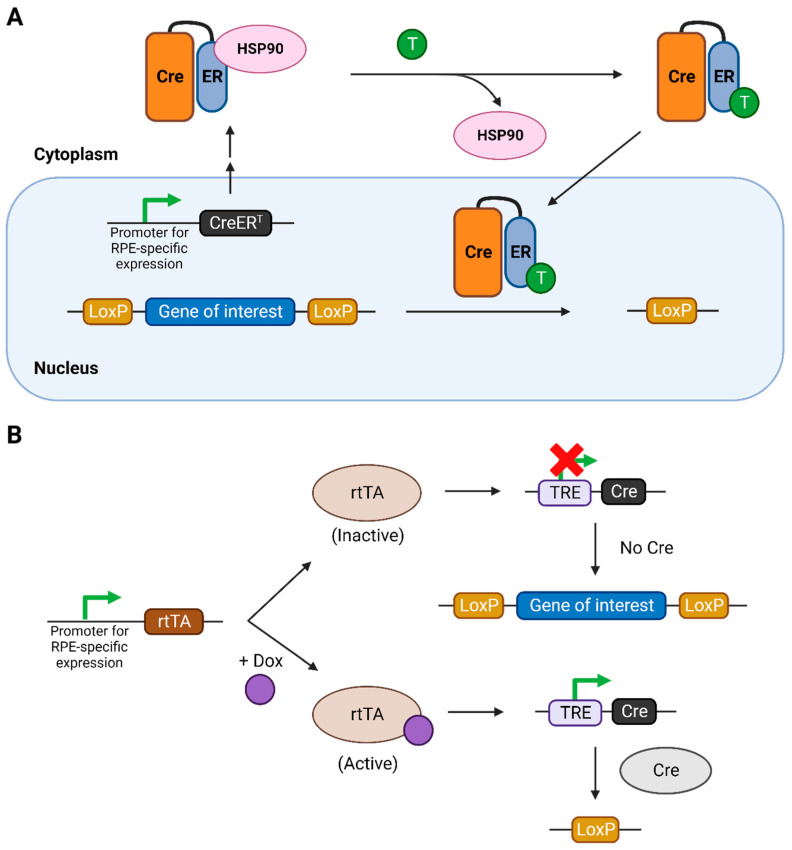
The inducible Cre-LoxP systems used for temporal regulation of the knockout of the gene of interest. (**A**) Tamoxifen-inducible Cre-LoxP system. (**B**) Tetracycline- or doxycycline-inducible Cre-LoxP system. Figure created using Biorender.com. Abbreviations: CreER^T^: fusion of Cre with modified ER ligand binding domain, ER: estrogen receptor, HSP: heat-shock protein, T: tamoxifen, rtTA: reverse tetracycline-controlled transactivator, TRE: tetracycline responsive element.

The tamoxifen-inducible system involves the use of a modified Cre recombinase, which includes its fusion with the estrogen receptor containing a mutated ligand binding site [19] under the control of a promoter expressed specifically in RPE cells (Figure 1A). This fused Cre protein is called CreER^T^ and is normally sequestered in the cytoplasm due to its binding with heat shock protein 90 (HSP90). Upon binding with synthetic steroids such as tamoxifen or its metabolically active form 4-hydroxytamoxifen (4-OHT), this interaction is severed and CreER^T^ translocates into the nucleus, acting on the LoxP sites, thereby knocking out the gene of interest. The efficiency of the system was later improved with the development of a modified version of CreER^T^, called CreER^T2^, which is roughly ten times more sensitive to tamoxifen or 4-OHT.

The tetracycline-inducible system involves the use of a reverse tetracycline-controlled transactivator (rtTA) protein, expressed specifically in RPE cells, and a tetracycline-responsive element (TRE) in the genome, which controls the expression of Cre recombinase (Figure 1B) [19]. Only upon binding with tetracycline, or its more efficient and cost-effective analogue doxycycline (dox), is the rtTA activated. This activation leads to its binding to the TRE, consequently activating the expression of Cre recombinase.

The tamoxifen system is generally preferred owing to its higher rate of induction [21,22] and specificity [23] among the inducible systems. Moreover, there may be trace amounts of tetracycline in mouse chow, leading to unintended ‘leaky’ expression of Cre recombinase. However, tamoxifen can have many undesirable side effects owing to its own biological activity [24,25,26]. Since it modulates the estrogen receptor, it may be advisable to thoroughly study sex-based differences in the induced mice before making final conclusions. Furthermore, there are concerns regarding the safety of its use, as prenatal exposure to tamoxifen is known to cause developmental defects in both humans [27,28] and mice [29,30]. Therefore, care should be taken with the dosage used to prevent any unwanted defects interfering with the phenotype to be studied and to limit any exposure to tamoxifen on the part of pregnant women involved in studies using these mice. The use of a doxycycline-inducible system is relatively safer compared to using tamoxifen owing to the limited toxicity of doxycycline to humans. It is to be noted that both these systems are to some extent susceptible to inducer-independent ‘leaky’ Cre recombinase activity [23,31], with the tetracycline-inducible system being more susceptible to it. Therefore, the Cre expression in these mice should be thoroughly validated before studies on the downstream effects of the gene knockout.

The expression of Cre recombinase specifically in the RPE of floxed mice can also be achieved via sub-retinal injection of viral vectors, like lentiviruses, containing expression elements for the enzyme in their genome. The specificity to the RPE is achieved due to the presence of the outer limiting membrane [32]. A similar injection technique has also been used to generate a CRISPR-based knockout of a gene of interest [33], where the gene sequences encoding both the Cas9 enzyme and the guide RNA (gRNA) are delivered using the viral vector. The main advantage of using such injection methods is that each researcher can generate a custom strategy to knockout any specific gene according to their research interests, which is relatively easier than generating new Cre mice. However, the use of this technique is limited by the requirement of expertise for sub-retinal injections, high resource and time investment owing to the high number of mice to be injected and relatively higher levels of variability. Since the strategies involving these techniques can be tailor-made for the needs of a researcher, this review will not discuss them further.

## 3. Non-Inducible Cre Mice Targeting the RPE

A summary of the various Cre mice discussed here can be found in Table 1. The selectivity and/or efficacy of the Cre mice can be monitored in 2 ways: (1) evaluating the expression levels of Cre protein by immunostaining or immunoblotting, or of Cre mRNA by (RT-q)PCR; (2) evaluating the function of the Cre enzyme by crossing the Cre mice with reporter mice like mT/mG or Rosa-lacZ [34,35,36,37], or by monitoring the knockout of the protein of interest. The mT/mG reporter mice express membrane-targeted tandem dimer Tomato (mT) in all cells of the body. In cells expressing Cre recombinase, upon Cre-mediated excision, membrane-targeted green fluorescent protein (mG) is expressed, allowing for a qualitative verification of Cre expression and function in these cells. The Rosa-LacZ mice, on the other hand, express beta-galactosidase upon Cre-mediated excision of an intervening segment in the gene. The presence and activity of this enzyme can be detected both in situ and in vitro via staining with X-gal, a chromogenic substrate of beta-galactosidase [38].

### 3.1. Tyrosinase Gene Family Promoters

The first Cre mice with an RPE-specific gene knockout were generated in 2002 [39,41]. These mice used promoters of genes specific to all pigmented cells (melanocytes and RPE) rather than to the RPE itself. These included the promoters of the genes from the tyrosinase gene family, tyrosinase-related protein 1 (*Trp1*) [39] and dopachrome tautomerase (*Dct*) [41]. Tyrosinase is the rate-limiting enzyme in the synthesis of melanin pigment from tyrosine [45]. The other enzymes in the family, tyrosinase-related protein 1 and dopachrome tautomerase, stabilize tyrosinase and melanosomes, along with carrying out their own functions in melanin synthesis [46,47].

Under the control of the *Trp1* promoter, Cre expression was observed from embryonic day (E10.5) to postnatal day 12 (P12) in the RPE [39]. However, there was ectopic expression in other ocular tissues, starting at various stages of development: the ciliary margin of the retina and the optic stalk at E11.5; a subset of cells in the ganglion cell layer (GCL) and neuroblastic layer of retina at E14.5; and the optic nerve, iris and ciliary body at P2-12. There was also ectopic expression in subsets of non-ocular cell types: the mesencephalon, the trigeminal nerve ganglion and the dorsal root ganglia.

Interestingly, there was no expression of Cre recombinase in other pigment cells, including those of the choroid. Similarly, a Cre mouse generated using the promoter for tyrosinase (*Tyr*) expressed Cre only in the melanoblasts of the skin and did not show expression in the RPE [48]. This led to the identification of regulatory elements that specify the expression of tyrosinase family genes *Trp1* and *Tyr* either to the RPE or to other melanocytes [49,50,51].

The adult *Trp1-Cre* mice showed no Cre expression in any of the tissues, suggesting that Cre expression was only temporary in these mice. This is inconsequential, as Cre-mediated genetic recombination is irreversible, and therefore, a transient exposure to this recombinase is sufficient to achieve a knockout of the gene of interest. Moreover, RPE cells are post-mitotic, which means that the cells with the gene of interest knocked out cannot be replaced over the lifetime of the mouse. However, despite such a transient expression, in a later study, these RPEs have been shown to suffer from Cre-mediated toxicity [40] in the form of reduced pigmentation, loss of RPE characteristic hexagonal shape and reduced thickness. The underlying retina was also affected, leading to reduced electroretinogram (ERG) responses and increased infiltration of microglia into the subretinal space. It is likely that this toxicity, despite a transient expression of Cre recombinase in these cells, arose from the toxic effects of Cre during the development of the RPE cells because RPE morphological defects were already evident at P14 [40].

The *Dct-Cre* mice, on the other hand, already showed Cre function in the optic cup, in dorsal telencephalon and in cells lateral to the neural tube at E9.5 [41]. Towards E12.5, the presumptive RPE, the migrating melanoblasts and the telencephalon showed Cre function, although not all the cells of these tissues showed the expression of the reporter gene. Owing to its Cre expression in melanoblasts, this Cre mouse has been used more in the context of pigment cell studies.

### 3.2. MART-1 Promoter

A different strategy, developed a decade later, also targeted the melanocytes by driving Cre expression using the promoter for the *MART-1* gene (melanoma antigen recognized by T cells 1), also called melan-A (*mlana*) [42]. It is thought to be essential for melanosome biogenesis [52]. These MART-1-*Cre* mice showed Cre expression in the RPE starting at E12.5 and later in the melanocyte precursors at E17.5. There was no ectopic Cre expression detected in these mice, except for some epidermal cells of the skin. No Cre toxicity was reported in these mice.

### 3.3. Bestrophin 1 Promoter

A Cre mouse more suitable for studying the effects of a gene knockout specifically in the RPE was later generated by making use of the promoter for the *Bestrophin 1* (*Best1*) gene [43], which codes for a calcium-activated chloride channel present on cell membranes [53]. It was found that loss of Bestrophin 1 function led to Best vitelliform macular dystrophy (BVMD) in humans, which resulted in the gene being also named the *VMD2* gene. In mice, Bestrophin 1 is specifically expressed only in the RPE cells, with the exception of Sertoli cells of the testes [43,54,55]. Concurrently, Cre expression in these mice was detected only in the RPE and the testes. Therefore, it may be prudent not to use male Cre-expressing mice for breeding, as their testes may be affected depending on the gene being knocked out.

Cre expression in the RPE of these mice starts from P10, with higher expression observed at P28. This circumvents the potential embryonic toxicity of Cre recombinase observed in the *Trp1-Cre* mice. However, owing to its continuous expression in the RPE cells from P10 onwards, the age- and dosage-dependent toxicity of Cre recombinase was later reported in the RPE of these mice [44], requiring a thorough examination of Cre-expressing controls. However, there appear to be inconsistencies in the literature with regard to such toxicity, as several reports using these mice, where no Cre-mediated RPE toxicity was observed, have been published [6,7,56,57].

Similar to the *Dct-Cre* mice, not all RPE cells in these mice express Cre recombinase, leading to what is termed a ‘mosaic pattern’ of Cre expression, which has also been observed in other Cre mice, that target different cell types [58,59]. It was later surmised to be a side effect of non-targeted integration of the Cre recombinase gene leading to the epigenetic silencing of the repeated copies of the inserted Cre gene [55,60,61,62]. The extent of RPE Cre expression depends on the strain and the age of the mice. While the C57BL/6 mice expressed Cre in 50–90% of their RPE cells, mice with a mixed B6/129 background showed consistent expression in 90% of their RPE cells. With respect to age, the percentage of RPE cells expressing Cre increased from ~15% at P10 to almost 70% by P28, peaking at ~90% at 9 weeks (9 w) of age. This mosaic expression of Cre recombinase in RPE cells can be taken advantage of, as this can provide an ideal internal control when Cre-expressing cells are identified. However, it comprises an impediment in biochemical studies like western blotting where individual Cre-expressing cells cannot be identified. In such cases, the percentage of RPE cells expressing Cre recombinase should be known. As it has been shown that recombination efficiency is similar in both eyes of a mouse, this information can be obtained by always using one eye to stain for Cre recombinase in the RPE [43,63], while the other can be used for any other experiment. This, therefore, limits the tissue availability of these mice. Despite these shortcomings, the *Best1-Cre* mice have since been extensively used to study the effects of conditional loss of various genes in the RPE [6,7,56,57,63,64,65,66].

## 4. Inducible Cre Mice Targeting the RPE

Inducible Cre systems have the advantage of letting the researcher decide when to knock out their gene of interest over the course of a mouse’s life span. This can bypass not only any potential developmental defects caused by the removal of the gene of interest during RPE development, but also any potential toxicity of the Cre recombinase function in developing RPE cells, which has been discussed in the previous section. A summary of the various Cre mice discussed in this section can be found in Table 2.

### 4.1. Tetracycline/Doxycycline-Inducible VMD2 Promoter

The first Cre mouse facilitating inducible RPE-selective gene knockout was the inducible VMD2-*Cre* model [67], which is regulated by tetracycline. Cre function could be induced as early as E9 until P60, with highest expression observed at P4. This covers the entire span of RPE development and therefore enables studying the importance of a gene during various stages of the process. However, these mice suffer from several drawbacks. Firstly, to achieve their maximum efficiency, they required tetracycline administration through gavage, which can only be conducted from P3 and needs relevant expertise. Secondly, the expression and function of Cre recombinase is not entirely inducer-dependent, meaning there is ‘leaky’ expression of Cre recombinase observed even when the mice are not administered tetracycline or its analogue, dox. Thirdly, these mice exhibited mosaic Cre expression that was revealed using reporter mice. This particular limitation is especially significant in these mice, as the levels of Cre recombinase could not be detected using immunohistochemical techniques. While these low expression levels could be good for avoiding any potential dosage-dependent Cre-induced toxicity to RPE cells, the inability to stain Cre recombinase precludes assessing the percentage of Cre-positive RPE cells. As pointed out before, the latter is important when using such mosaic Cre-expressing RPE for biochemical studies. Finally, these mice also showed some ectopic Cre recombinase function in the optic nerve cells. Despite these shortcomings, the *VMD2-Cre* mice were quite useful at the time, being one of the few models that did not suffer from Cre–induced RPE toxicity, with intact RPE and retinal integrity and function even up to 10 months of age.

To tackle some of the drawbacks of these mice, the authors later evaluated the efficacy of administering dox via intravitreal injections [69]. This allowed for better efficiency of Cre expression and function, with ~60% of the RPE showing Cre activity starting from 15 days post injection. No Cre levels were detected at 4 months post injection, which is beneficial in avoiding any potential Cre-induced toxicity. Indeed, the retinal morphology was preserved even up to 12 months after dox induction. This also prevented the ectopic expression of Cre recombinase in the optic nerve, which was observed when dox was administered orally. However, certain drawbacks remain with regard to the technical expertise required for intravitreal injections, especially in neonates, and the fact that it is not possible to perform this procedure in embryonic mice.

### 4.2. Tamoxifen-Inducible MCT3 Promoter

Around the same time, a tamoxifen-inducible RPE-selective gene knockout mouse was generated using the promoter for the *Slc16a8* gene, which codes for monocarboxylate transporter 3 (MCT3) [5]. The MCT3 protein is responsible for transport of several monocarboxylate substrates, including lactate, pyruvate and some ketone bodies, across the cell membrane. It is only expressed in the RPE and in the choroid plexus epithelium of the brain [72,73]. Concurrently, the MCT3-*Cre* mice exhibited Cre function only in these two cell types without any unexpected ectopic expression. In neonates, Cre function was detected in the RPE 7 days post injection of 4-OHT. However, they also suffer from mosaic Cre activity, with only ~20% of the RPE cells exhibiting Cre function, which further fell to ~5% when older mice were induced. Owing to their very low efficiency, these mice were not widely used.

### 4.3. Tamoxifen-Inducible Trp1 Promoter

Soon after, another inducible Cre mouse was generated using the promoter for the *Trp1* gene paired with the tamoxifen-inducible Cre system. A week after induction with tamoxifen, Cre activity was detected in the RPE in a mosaic pattern, with ~40–80% of the RPE exhibiting Cre-mediated recombination. Interestingly, in contrast to the *Trp1-Cre* mice [70], no choroidal or extra-ocular Cre recombinase activity was detected using these inducible *Trp1-Cre* mice. However, there was still some ectopic Cre function detected in some cells of the neural retina, iris, ciliary body and optic nerve.

### 4.4. Tamoxifen-Inducible hsp70 Promoter with RPE-Specific Cns-2 Enhancer

Schneider et al. generated an inducible RPE-selective Cre mouse by expressing tamoxifen-inducible Cre-ER^T2^ under the control of ubiquitous *hsp70* minimal promoter and RPE-specific *Tyr* enhancer (*Cns-2*) [22]. RPE specificity was conferred by the *Tyr* enhancer element *Cns-2*, which was shown to drive tyrosinase expression specifically in the RPE cells [51].

Cre activity was only detected upon induction with 4-OHT in the RPE and to some extent in the ciliary body (~10%). Some ectopic Cre activity was detected in the inner nuclear layer (INL) of the neural retina, but this was not specific to any cell type in the retina. No extra-ocular Cre activity was detected in these mice. In adult mice, after 5 consecutive days of tamoxifen treatment, ~47–70% of the RPE exhibited Cre recombinase activity, with better efficiency observed in the central RPE compared to the peripheral RPE. Similar to inducible *MCT3-Cre* mice, the efficiency of tamoxifen induction increased when embryonic mice were induced at E9.5 (through the mother) for 5 days, with the percentage of RPE showing Cre activity rising to ~83%. This difference in efficacy was attributed to the possible differences in the effective dosage of tamoxifen to the RPE and a possibly higher efficacy of recombination in the developing RPE as opposed to a fully differentiated adult RPE. Interestingly, some sex-based differences in the efficacy of the tamoxifen administration method (Intraperitoneal (IP) vs. gavage) were also reported, with the IP route being more efficient in males than in females. No Cre toxicity was reported even at 3 months post induction. However, this was only assessed in adult mice induced with tamoxifen. Any potential toxicity of Cre expression when induced in the embryonic RPE was not evaluated and thus needs to be thoroughly examined before conclusions are drawn regarding these mice.

### 4.5. Tamoxifen-Inducible Best1 Promoter in Rosa26 Locus

Since most mice with RPE-selective gene knockout suffer from mosaic Cre activity, Chen et al. tackled this by targeting the insertion of the *Best1* promoter-controlled transgenic *Cre-ER^T2^* gene into the *Rosa26* locus of C57BL/6J mice [55]. Despite such targeted insertion, there was still some mosaic activity of Cre recombinase, suggesting that the mosaicism could arise from an intrinsic feature of the *Best1* promoter. Cre recombinase activity was most efficiently induced via 4 consecutive daily IP injections of 4-OHT beginning at P14, with no leaky expression observed. While ~90% of RPE cells in males exhibited Cre activity, the percentage was lower in females at ~85%. Robust induction was also observed in adult mice at 7 w of age with ~85% percent of the RPE showing Cre activity in both genders. However, this required double the dosage that was used at P14. No Cre activity was detected in any other tissue except for the testes, which is expected based on the expression pattern of the *Best1* gene in mice. Minimal/negligible Cre activity was detected in the Müller glia of the retina, which never exceeded 0.6% of these cells. This is not entirely surprising because the *Best1* promoter has been shown to drive Cre expression in Müller cells before [68]. Moreover, no RPE or retinal toxicity was observed even at 7 months of age in the mice induced at P14. However, the same could not be said regarding the homozygous Cre mice, which showed RPE morphological abnormalities at 8 months. The heterozygotes were normal until 2 y, and therefore, it is judicious to only use these. This dose-dependent difference in Cre toxicity is in line with previous observations seen in non-inducible *Best1-Cre* mice [43,44]. The consistently high percentage of RPE cells expressing Cre recombinase in these mice eliminates the requirement for the use of one eye to assess the percentage of Cre-expressing RPE cells in a given mouse, leaving both the eyes for experiments of choice to the researcher.

### 4.6. Tamoxifen-Inducible Cre Recombinase Gene Conjugated to Rpe65 Gene

During the time of development of the inducible *Best1-Cre* mice, the generation of another Cre mouse was underway, which largely eliminated many of the limitations of the previously generated mice. This mouse takes advantage of the expression pattern of the native *Rpe65* gene [62], which encodes one of the most important visual cycle proteins specific to RPE cells. It catalyzes the conversion of all-*trans*-retinyl esters to 11-*cis*-retinol, which is then converted to regenerate 11-cis-retinal, the visual chromophore crucial for vision [3]. Instead of creating a traditional transgenic mouse, the authors knocked in (KI) a *P2A-CreER^T2^* sequence, fused in-frame after the last coding exon of the native *Rpe65* gene. P2A is a nucleotide sequence that causes efficient ribosome skipping during protein translation [74,75], allowing for the production of 2 different proteins from a single transcript: the RPE65-P2A fusion protein, appended on its C-terminal end with non-native 21 amino acids; and the Cre-ER^T2^ protein [62]. Unfortunately, the addition of C-terminal non-native amino acids to the RPE65 protein led to its reduced stability, as observed from a ~40% reduction in the levels of RPE65 in these mice. This reduction was much more pronounced (~99%) in homozygous KI mice. Therefore, it is again advisable to only use the heterozygous mice. To test the efficacy of *Rpe65-Cre-ER^T2^* mice, tamoxifen was administered either via IP injections (5 consecutive days) or via chow (3 weeks) at P21 and P50. Negligible Cre activity was detected in the absence of tamoxifen induction. In the induced mice, ~99% of the RPE cells showed Cre activity, with the non-expressing cells predominantly present in the peripheral retina. No ectopic expression of Cre recombinase was detected. The levels of Cre recombinase were below the detection limit for immunoblotting. Concurrently, there was no Cre-induced toxicity detected in the RPE and the retina of these mice even at the age of 4 months. However, in view of the reduced expression of RPE65 protein, the retinal function was tested. This was found to be unaffected at P30, but the rate of 11-*cis*-retinal regeneration was reduced and all-*trans*-retinyl esters accumulated, especially in mice carrying the less active M450 allele of *Rpe65* gene as compared to the more efficient L450 allele. While this was not significant at P30, any potential changes in the retinyl esters or 11-*cis*-retinal regeneration at later ages were not reported. However, any such potential changes at later ages can be presumed to be minimal, as no significant differences were observed in the electroretinograms of 4-month-old mice, showing that the retinal function was intact. One limitation with these mice involves native RPE65 expression, which only starts after P4 in rats, which is likely the case in mice as well [76]. Therefore, induction of gene knockout before this time is likely not possible using these mice. Moreover, the authors only induced Cre activity from P21 onwards, by which time the RPE is fully developed. This likely circumvents any potential toxicity of Cre function to the developing RPE. However, if Cre function is to be induced at time points before P21, it is prudent to thoroughly characterize the integrity of the RPE and the retinas of these mice.

### 4.7. Tetracycline/Doxycycline-Inducible Pmel Promoter

Most recently, another Cre mouse model has been generated using the tetracycline-inducible *Pmel* gene promoter, which codes for premelanosome protein [71], which is important in the early stages of melanosome biogenesis. These mice were generated with the purpose of obtaining an inducible gene knockout not just specifically in the RPE, but in all the melanocytes. Dox was administered to pregnant mice through drinking water for 3 days at 10.5 days post coitus. The embryos were assessed for Cre activity at E15.5. Cre activity was detected in all pigment cells, as expected from the expression pattern of the *Pmel* gene. There was some ectopic expression in the mesothelial cells of the heart and the lungs, which was independent of exposure to dox (‘leaky expression’). Interestingly, the authors did not report mosaic Cre expression in these mice despite a non-targeted integration ap proach to insert the transgene. Very little has been reported on the RPE of these mice, and since they have not yet been used for studies involving RPE-specific gene knockout, the advantages of these mice over others for such a purpose are less clear.

## 5. Conclusions

The availability of the various Cre mice that enable an RPE-specific gene knockout provides several options for researchers to achieve their research goals. It is impossible to recommend a single Cre mouse line for all purposes, and the choice needs to be made based on the specific research question of interest to the investigator. While the *Best1-Cre* mouse line [43] remains the most used among the non-inducible Cre lines due to its postnatal start of Cre expression and minimal ectopic expression of the recombinase, it still suffers limitations in the form of mosaic Cre expression and age-dependent toxicity. Among the inducible Cre lines, the more recently developed inducible *Best1-Cre* [22] and *Rpe65-Cre* [62] lines are promising, as they largely eliminate mosaic Cre expression, which plagues most models, and they do not exhibit Cre toxicity. However, the *Rpe65-Cre* line requires further validation with regard to the onset of Cre expression.

With the advent of new tools, in particular CRISPR-Cas9 methodologies, it can be expected that the research portfolio will be expanded. Still, the Cre-LoxP system will remain essential in years to come in view of technical requirements of the CRISPR-based methods. It is important to note that in order to study the intricate molecular pathways and interactions between various macromolecules at the subcellular level, and to study various functional parameters such as the mitochondrial oxygen consumption rate, researchers will need to adopt the usage of in vitro models in addition to in vivo models.

## Figures and Tables

**Table 1:** RPE-selective Cre-expressing mice without an inducible Cre system.

Model	Promoter	Cre Expression Start	Important Features	References
*Trp1-Cre*	Tyrosinase-related protein 1 (*Trp1*) promoter	E10.5	- Untargeted insertion of Cre - Cre toxicity to RPE - Ectopic Cre expression in some cells of neural retina, along with some other tissues	[39,40]
*Dct-Cre*	Dopachrome tautamerase (*Dct*) promoter	E9.5	- Untargeted insertion of Cre - Mosaic Cre expression - Cre expression also in melanocytes and in cells of the telencephalon * - Ectopic Cre expression in caudal nerves and dorsal root ganglia	[41]
MART-1-*Cre*	Melanoma associated antigen recognized by T-cells (MART-1) promoter	E12.5	- Untargeted insertion of Cre - Uniform Cre expression - Cre expression also in all melanocytes * - Minimal ectopic Cre expression in some epidermal cells of the skin	[42]
*Best1-Cre*	Bestrophin-1 (*Best1*) promoter	P10	- Untargeted insertion of Cre - Mosaic Cre expression - Age- and dosage-dependent Cre toxicity to RPE - Cre expression also in testes *	[43,44]

* Cre expression in these cells is expected based on the promoter expression pattern. Contradictory data on Cre toxicity were reported.

**Table 2:** RPE-selective Cre-expressing mice with an inducible Cre system.

Model	Promoter	Induction by	Important Features	References
Inducible VMD2-*Cre*	Vitelliform macular dystrophy-2 (VMD2), promoter	Tetracycline/Doxycycline	- Untargeted insertion of Cre - ‘Leaky’ Cre expression - Mosaic Cre expression - Cre recombinase undetectable by immunostaining - Weak ectopic Cre expression in the optic nerve	[67,68,69]
Inducible MCT3-*Cre*	Monocarboxylate transporter 3 (MCT3) promoter	Tamoxifen	- Untargeted insertion of Cre - Mosaic Cre activity; only 5–20% of RPE show Cre activity - Cre activity also in the choroid plexus epithelium of the brain *	[5]
Inducible *Trp1-Cre*	Tyrosinase-related protein 1 (*Trp1*) promoter	Tamoxifen	- Untargeted insertion of Cre. - Mosaic Cre activity - Ectopic Cre activity in some cells of neural retina, iris, ciliary body and optic nerve	[70]
Inducible *Tyr-Cre*	Tyrosinase (*Tyr*) promoter	Tamoxifen	- Untargeted insertion of Cre - Mosaic Cre activity with better expression in embryonic RPE - Cre activity also observed in the ciliary body * - Weak ectopic Cre function observed in inner nuclear layer without any cell-type specificity	[22]
Inducible *Best1-Cre*	Bestrophin-1 (*Best1*) promoter	Tamoxifen	- Targeted insertion of Cre gene into the Rosa26 locus - Cre function also in the testes * - Cre activity in >90% of RPE cells - Minimal/negligible (<1%) ectopic Cre function in Muller glia	[55]
Inducible *Rpe65-Cre*	Retinal pigment epithelium-specific 65 kDa protein (*Rpe65*) promoter	Tamoxifen	- Targeted knock-in of sequence for P2A-CreERT2 fused in-frame with *Rpe65* gene - Cre activity in >90% of RPE cells - Levels of Cre recombinase undetectable by immunoblotting	[62]
Inducible *Pmel-Cre*	Premelanosome protein (*Pmel*) promoter	Tetracycline/Doxycycline	- Untargeted insertion of Cre. - No mosaic Cre expression - Cre expression also in most melanocytes * - Ectopic Cre expression in lung and heart mesothelial cells	[71]

* Cre expression in these cells is expected based on the promoter expression pattern.

## Data Availability

Not Applicable.
